# Multisystem Inflammatory Syndrome in Children (MIS-C) in a Lithuanian Paediatric Tertiary Care Center

**DOI:** 10.3390/medicina60111774

**Published:** 2024-10-30

**Authors:** Indrė Stacevičienė, Inga Ivaškevičienė, Odeta Kinčinienė, Loriana Kilaitė, Augustina Jankauskienė

**Affiliations:** Faculty of Medicine, Vilnius University, 03101 Vilnius, Lithuania; inga.ivaskeviciene@mf.vu.lt (I.I.); odeta.kinciniene@mf.vu.lt (O.K.); loriana.kilaite@mf.vu.lt (L.K.); augustina.jankauskiene@mf.vu.lt (A.J.)

**Keywords:** MIS-C, severity, intensive care unit (ICU), cardiac involvement, echocardiography

## Abstract

*Background and Objectives*: Due to its link with the SARS-CoV-2, Multisystem Inflammatory Syndrome in Children (MIS-C) gained global attention as a serious condition that requires hospital care. Our study aimed to present the clinical and laboratory characteristics of MIS-C patients by age group and intensive care unit (ICU) admission status and assess early echocardiographic changes. *Materials and Methods*: A single-center partly retrospective, partly prospective observational cohort study was performed from December 2020 to June 2024. The study included 42 patients aged between 1 month and 18 years who were diagnosed with MIS-C and gave informed consent. *Results*: The median age was 6.5 years (IQR 2.0–9.3). The predominant symptoms were cardiovascular (88.1%), mucocutaneous (85.7%) and gastrointestinal (76.2%). Five children (11.9%) developed shock. About two-thirds of patients (66.7%) were admitted to the ICU. Adolescents (≥12 years) were less likely to exhibit mucocutaneous or cardiovascular symptoms and thus less frequently having Kawasaki—like disease symptoms compared with other age groups (<5 years or 5–11 years). Lymphopenia was more common among patients aged 5 years and older. Adolescents had higher procalcitonin (PCT) and a lower estimated glomerular filtration rate. Troponin I and B-type natriuretic peptide (BNP) levels were higher in children aged 5–11 years, while ferritin levels were lower among the youngest (<5 years). Patients treated at the ICU were more likely to have cardiovascular and respiratory symptoms, as well as a history of symptomatic COVID-19, higher C-reactive protein (CRP), PCT, BNP and lower albumin levels. Echocardiographic abnormalities were found in 71.4% of cases. During hospitalization, left ventricular ejection fraction values increased significantly (*p* < 0.001) over 12 (IQR 9.0–14.0) days. *Conclusions*: Symptoms and laboratory markers of MIS-C vary according to age. Higher CRP, PCT, BNP and hypoalbuminemia are predictors of MIS-C severity. Cardiovascular involvement is common and might be severe, but rapid resolution is encouraging.

## 1. Introduction

First described in the United Kingdom in April 2020 [1], Multisystem Inflammatory Syndrome in Children (MIS-C) was soon identified in many countries, gaining global attention as a serious condition that requires hospital care [2,3]. Lithuania also encountered this problem, but the first case was diagnosed relatively late, at the end of 2020, therefore officially registered only in 2021 [4]. Later, the number of cases increased, and MIS-C became part of routine clinical practice.

MIS-C is characterized by fever, elevated inflammatory markers, evidence of SARS-CoV-2 infection, and multisystem organ involvement, with no other alternative diagnosis [5,6]. The syndrome can present with a wide range of clinical features, affecting the gastrointestinal, mucocutaneous, cardiovascular, respiratory, hematological, neurological and renal systems [7,8,9,10]. Diagnosing MIS-C requires thorough investigation, including a series of laboratory and instrumental tests. One of the most challenging aspects of managing MIS-C is its impact on the cardiovascular system, which necessitates extensive testing and ongoing monitoring [11,12,13].

The syndrome primarily affects children and young adults up to 21 years, with the highest risk of MIS-C between the ages of 5 and 11 years [9,14]. Limited data are reported regarding age differences in the presentation. The severity varies from milder cases to severe complications requiring intensive care [9,10]. Around half or more of MIS-C patients typically require admission to an intensive care unit (ICU), and the mortality rate can reach up to 5% [8,9,10,15,16,17]. There is variability in the data regarding factors associated with severe outcomes [8,11,16,17,18]. Our study aims to present the clinical and laboratory characteristics of MIS-C patients by age group and ICU admission status and assess early echocardiographic changes in a single large tertiary care center.

## 2. Materials and Methods

A single-center, partly retrospective, partly prospective observational cohort study was performed from December 2020 to June 2024. Patients were recruited at the Vilnius University Hospital Santaros Klinikos, designated as a major hospital for admissions of patients with MIS-C in the northeast region of Lithuania. This study was approved by the Vilnius Regional Biomedical Research Ethics Committee (No. 2022/3-1419-888) and conducted in accordance with the principles of the World Medical Association Helsinki Declaration as well as local law.

The study included patients from 1 month to 18 years who met the national diagnostic criteria of MIS-C [19], adapted from the WHO and CDC [5,6]: fever ≥ 38.0 °C for ≥24 h; new onset manifestations in at least two different organ systems (gastrointestinal, mucocutaneous, cardiovascular, respiratory, renal, neurological and hematological); evidence of COVID-19 (RT-PCR or serology positive); laboratory evidence of inflammation; exclusion of an alternative diagnosis.

Patients were excluded if there was no written informed consent form.

Detailed data of each patient were obtained from patient electronic medical records:

- basic characteristics (age, gender, race, height, weight, date of hospitalization);
- epidemiological data (known previous COVID-19 infection, vaccination status against SARS-CoV-2);
- clinical data (gastrointestinal, mucocutaneous, cardiovascular, respiratory, renal, neurological and other symptoms);
- comorbidities;
- laboratory data (SARS-CoV-2 RT-PCR or serology, full blood count (FBC), C-reactive protein (CRP), procalcitonin (PCT), interleukin 6 (IL-6), ferritin, troponin I, B-type natriuretic peptide (BNP), D-Dimer, albumin and estimated glomerular filtration rate (eGFR, which was based on the revised Schwartz equation [20]);
- echocardiographic initial findings and early outcome on discharge (left ventricular ejection fraction (LVEF), coronary artery involvement, valvular regurgitation, pericardial effusion);
- electrocardiographic (ECG) findings (conduction abnormalities such as atrioventricular (AV) block, bundle branch blocks; tachyarrhythmias, including premature beats; significant repolarization abnormalities, including ST deviation > 1 mm, negative T waves at I, II, aVF, V5-6; QTc prolongation);
- treatment (oxygen therapy, vasoactive drugs, intravenous immunoglobulin (IVIG), glucocorticoids, other immunomodulators);
- outcome data (duration of hospitalization, admissions to ICU, recovered/deceased).

Clinical data were defined by selecting the most prominent signs and symptoms: gastrointestinal (abdominal pain, diarrhea, vomiting, abnormal liver function tests, colitis, ileitis and ascites), mucocutaneous (conjunctivitis, periorbital swelling/redness, mucus membrane changes, strawberry tongue, rash, lymphadenopathy, swollen hands and feet), cardiovascular (tachycardia, high blood pressure, arterial hypotension, shock), respiratory (cough, sore throat, oxygen requirement, patchy infiltrates, pleural effusion), neurological (headache, confusion, irritability, reduced level of consciousness/lethargy, syncope), renal (renal function impairment, decreased diuresis, urine sedimentation abnormalities), other (arthralgia, myalgia).

Based on the clinical presentation, MIS-C patients were divided into three subgroups [7] as follows: MIS-C with shock; Kawasaki—like disease (KD; patients with fever, lymphadenopathy, mucocutaneous and cardiovascular involvement); undifferentiated MIS-C (patients with fever and inflammation who did not meet either KD criteria or symptoms of shock).

Nasopharyngeal swabs were tested using real-time reverse-transcriptase polymerase chain reaction (RT-PCR) tests for SARS-CoV-2, using the Xpert Xpress plus (Cepheid, Sunnyvale, CA, USA) with 100% specificity and 100% sensitivity. COVID-19 serology was tested by quantification of SARS-CoV-2 anti-RBD IgG antibodies against the RBD domain of the spike protein.

Cardiac echocardiography was performed by two specialists using an ultrasound system Samsung HS7A74L/WR (Suwon-si, Republic of Korea). A normal LVEF was defined as an LVEF of 55% or higher. Coronary artery involvement was classified according to the American Heart Association (AHA) guidelines, using the z-score system: z-score < 2—no involvement; z-score ≥ 2 to <2.5—dilation; z-score ≥ 2.5—aneurysm [21]. AV or other valve regurgitation or pericardial effusion were deemed abnormal or not on the basis of the echocardiography report.

Categorical data were presented as frequencies and percentages and analyzed using Fisher’s exact test. For continuous data, medians/interquartile range (IQR) were calculated. A one-way analysis of variance (ANOVA) was used to compare the three age groups (<5 years, 5–11 years and >12 years) with possible covariates. Initial and follow-up echocardiographic findings were compared using McNemar’s test for categorical variables and paired-*t*-tests for continuous variables. Student’s *t*-test and Kruskal–Wallis H test were used for categorical–continuous variable pairs. Statistical analyses were performed with IBM SPSS Statistics 22.0 (Chicago, IL, USA). A *p* value < 0.05 was considered significant.

## 3. Results

### 3.1. Study Sample

A total of 51 patients have been diagnosed with MIS-C over 3.5 years. Forty-two children were included in the final analysis. Nine patients were excluded as their parents did not provide written informed consent. Of these, three patients with comorbidities died.

**Figure 1:**
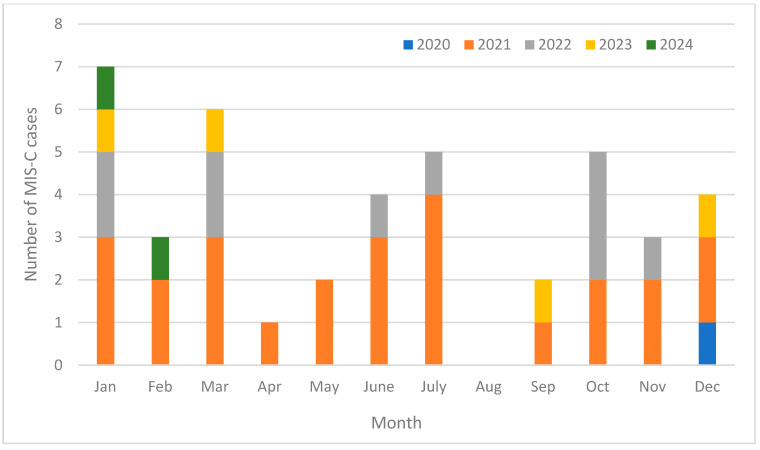
Monthly distribution of MIS-C, December 2020–June 2024.

The first case of MIS-C was diagnosed in December 2020. The majority of cases were in 2021 (*n* = 25) with a declining number of cases in subsequent years (ten cases in 2022, four cases in 2023 and two cases during the first half of 2024). The monthly distribution of cases showed slight increases in January and March, while August had no cases at all (Figure 1).

All enrolled patients were Caucasian, with more boys (73.8%, *n* = 31) than girls. The youngest patient was 9 months old, and the oldest was 16 years. The median age was 6.5 years (IQR 2.0–9.3). All patients had fever. The predominant symptoms were cardiovascular (88.1%, *n* = 37), mucocutaneous (85.7%, *n* = 36) and gastrointestinal (76.2%, *n* = 32). Eight patients (19.1%) had two organ systems involved. About two thirds of patients (69.0%, *n* = 29) had symptoms involving three or four organ systems, while the remaining five patients (11.9%) had more extensive involvement.

The most common MIS-C phenotype was KD, seen in 73.8% of cases (*n* = 31). Five children had shock symptoms (11.9%), and six children presented with undifferentiated MIS-C (14.3%). Adolescents (≥12 years) were less likely to exhibit mucocutaneous or cardiovascular symptoms and thus less frequently having KD symptoms compared with other age groups (<5 years or 5–11 years; Table 1).

The majority of patients presented with leucocytosis (>12 × 10^9^/L; 69.0%), neutrophilia (>7.5 × 10^9^/L; 78.6%) and lymphocytopenia (<1.5 × 10^9^/L; 44.1%). Thrombocytosis (>450 × 10^9^/L) was more common than thrombocytopenia (<140 × 10^9^/L; 40.0% and 27.5%, respectively). All inflammatory markers increased, most notably CRP and IL-6: 76.2% (32/42) patients had CRP >100 mg/L and 52.2% (12/23) had IL-6 >100 ng/L. An increase in BNP was higher and more common compared to troponin I: 62.9% (22/35) had BNP > 100 ng/L and 41.0% (16/39) had troponin I > 16 ng/L. Most patients had significantly elevated ferritin and D-dimer levels: ferritin > 300 μg/L in 63.4% (26/41) and D-dimer > 1000 μg/L in 80.5% (33/41) of cases. Hypoalbuminemia (<38 g/L) was seen in 84.8% (28/33) of patients and reduced eGFR (<90 mL/min/1.73m^2^)—in 29.3% (12/41) of cases.

Laboratory tests revealed age-related differences in several parameters (Table 1). Lymphopenia was more common among patients aged 5 years and older. Adolescents (≥12 years) had higher PCT and lower eGFR. Troponin I and BNP levels were higher in children aged 5–11 years, while ferritin levels were lower among the youngest (<5 years).

None of the patients were vaccinated against COVID-19. Almost two-thirds (62.8%, *n* = 27) of the patients were not vaccinated, as there was no age-appropriate SARS-CoV-2 vaccine at the time. Data about eight patients, who could have been vaccinate were missing.

All tested children (*n* = 41) had a positive SARS-CoV-2 serology. One patient was not tested but had a recent history of COVID-19. Additionally, thirty-eight children were tested for SARS-CoV-2 PCR, and the test was positive in six cases (14.3%).

### 3.2. Echocardiography Findings

All children included in the analysis underwent cardiac ultrasound at least twice. We compared initial echocardiographic findings with discharge data. A follow-up echocardiogram was performed with a median of 12.0 (IQR 9.0–14.0) days after the initial echocardiogram.

**Figure 2:**
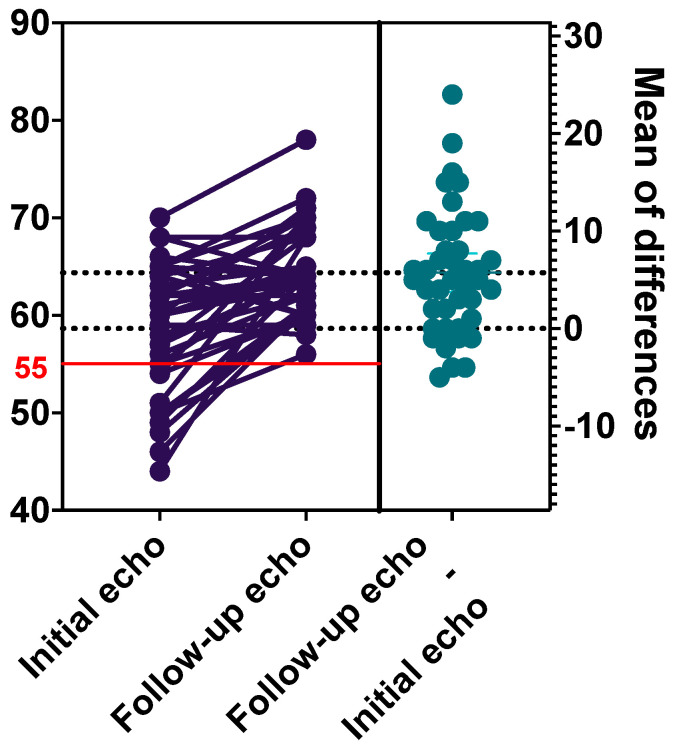
Left ventricular systolic dysfunction (LVEF): initial data and early follow-up changes at hospital discharge. A normal LVEF was defined as an LVEF of 55% or higher. The left side shows LVEF initial findings and follow-up changes at hospital discharge. The right side shows the mean of LVEF differences.

Initially, 30 patients (71.4%) had echocardiographic abnormalities. The most common findings were pericardial effusion (64.3%, *n* = 27) and valvular regurgitation (54.8%, *n* = 23). Atrioventricular valve regurgitation was the most common (47.6%, *n* = 20). Mitral valve and tricuspid valve regurgitation was described in 31.0% of cases (*n* = 13). Pulmonary valve regurgitation was detected in 14.3% (*n* = 6) and aortic valve regurgitation—in 4.8% (*n* = 2). A decreased LVEF <55% was found in 26.2% patients (*n* = 11). There were no coronary artery aneurysms, only one patient had coronary artery dilation. Echocardiographic parameters improved during early follow-up (Table 2), including LVEF values, which significantly increased during the treatment (*p* < 0.001; Figure 2).

### 3.3. Treatment and Outcome Data

Children were hospitalized from 5 to 43 days. The average duration of hospitalization was 13 days (IQR 9.75–15.25). About two-thirds of patients (66.7%, *n* = 28) were admitted to ICU and treated there for 2 to 8 days. The average duration in ICU was 4 days (IQR 3.0–5.0). Patients treated at the ICU were more likely to have cardiovascular and respiratory symptoms, as well as a history of symptomatic COVID-19, higher CRP, PCT, BNP and lower albumin levels (Table 3). The percentage of comorbidities was the same in both ICU and non-ICU patients.

Most patients were treated with IVIG (92.9%, *n* = 39) and GCC (81.0%, *n* = 34). A combination of IVIG and GCC was given to 32 patients (76.2%), 7 patients received IVIG alone, 2 patients received GCC alone and 1 patient received only symptomatic treatment. Eleven patients (26.2%) required oxygen therapy, which lasted on average from 1 to 5 days. Vasoactive drugs were administered also from 1 to 5 days in five children (11.9%). Treatment differences between age groups and ICU/non-ICU patients are shown in Table 1 and Table 3. All study patients were discharged.

## 4. Discussion

This study covers the entire period of MIS-C history in our pediatric center, as it analyses data from the first case of MIS-C in 2020 up to summer 2024. A notable peak in MIS-C cases occurred in 2021, coinciding with the prevalence of the SARS-CoV-2 Alpha and Delta variants in Europe [22]. Subsequent years showed a gradual decline in cases, mirroring trends observed internationally [23,24], possibly due to increased community exposure to other COVID-19 variants like Omicron and rising vaccination rates [23,25,26,27].

While some regions reported a lag between COVID-19 peaks and subsequent rises in MIS-C cases [28,29,30], our data did not show any clear seasonal trends or direct correlations with spikes in COVID-19 infections. For instance, despite a surge in SARS-CoV-2 infections among children in our hospital from January to March 2022 [31], MIS-C cases remained scattered throughout the year.

Our cohort was exclusively Caucasian, preventing any assessment of racial influences on MIS-C, although it is known that Black and Hispanic children appear to be disproportionally affected, while Asian children account for only a small number of cases [32]. Males predominated, consistent with global observations [9,25,33]. Sex differences are possibly due to heightened immune responses in males leading to more severe inflammatory reactions [33]. The median age of the MIS-C patients was 6.5 years, aligning with global data [7,15,17,25], and we observed notable clinical and laboratory differences across age groups. For example, lymphopenia was less common in the youngest cohort (under 5 years), who also exhibited lower ferritin levels. In contrast, school-aged children (5–11 years) showed elevated levels of Troponin I and BNP, indicative of more significant cardiovascular involvement—a potential factor in the higher ICU admission rates observed in this age group. Other studies have also pointed out that children of this age are at higher risk of being admitted to the ICU [16,17].

Adolescents (12 years and older) were less prone to mucocutaneous or cardiovascular symptoms and abnormal echocardiographic findings but exhibited higher PCT rates and reduced eGFR. Most of the cases with reduced eGFR were of prerenal cause and lasted just a few days till fluid balance was restored [34]. Adolescents showed fewer Kawasaki-like symptoms, in accordance with other reports [10]. This may be related to the fact that classic KD typically affects infants and young children [35].

Severity predictors for MIS-C are diverse. Data suggest that symptoms such as shortness of breath, abdominal pain, seizures and renal failure are more likely to precipitate the ICU admissions [8,16,17]. In our study, patients requiring treatment at the ICU more frequently presented with cardiovascular and respiratory symptoms and demonstrated higher CRP, PCT, BNP, and lower albumin levels—trends consistent with global observations [11,16,17,18]. Furthermore, a constellation of abnormal laboratory markers, including reduced platelet or lymphocyte counts, increased troponin, ferritin, D-dimer, fibrinogen and IL-6 were identified in other studies as correlates of ICU necessity, emphasizing that both inflammatory and cardiac markers serve as crucial severity predictors for MIS-C [8,11,16,17,18]. In our study group, comorbidities did not have a clear impact on the course of the disease, except that we cannot speculate about excluded patients who had comorbidities and died.

Cardiovascular involvement, a critical concern in MIS-C, was prominent among our patients, with a majority exhibiting echocardiographic abnormalities. ICU patients commonly showed heightened cardiovascular symptoms and higher BNP levels. Fortunately, cardiac abnormalities in MIS-C cases tend to resolve swiftly, as indicated by the significant improvement in our echocardiographic findings at discharge, although some patients showed lingering issues such as valve regurgitation and pericardial effusion. It is hypothesized that myocardial injury in MIS-C results from transient myocardial edema caused by intense inflammation rather than direct viral damage, with most cases of LVEF normalizing within 1 to 2 weeks [12,13,15,36]. However, MRIs performed several months post-diagnosis revealed persistent cardiac abnormalities in some cases [37], highlighting the need for extended follow-up.

Our study has few limitations, as it is a single-center study with a relatively small number of patients, but the results are presented as real data and reflect the situation in Eastern Europe. Another limitation is that patients who died were not included in the study because the parents did not give permission.

## 5. Conclusions

This study illustrates variability in MIS-C symptoms and laboratory markers across different age groups and identifies severity predictors—higher CRP, PCT, BNP and hypoalbuminemia. Our data confirm that focus on the cardiovascular disorders among MIS-C cases is essential as they are quite common and might be severe. While the rapid resolution of cardiac abnormalities post-treatment is encouraging, long-term follow-up and continuous research at both national and international levels remain crucial.

## Figures and Tables

**Table 1:** Demographic and clinical characteristics at admission of MIS-C patients by age group.

	All Cases	<5 Years	5–11 Years	≥12 Years	*p*-Value
Counts (%)	42 (100)	18 (42.9)	18 (42.9)	6 (14.3)	
Male sex	31 (73.8)	13 (72.2)	13 (72.2)	5 (83.3)	0.858
Comorbidities	9 (21.4)	3 (16.7)	3 (16.7)	3 (50.0)	0.193
A known history of symptomatic COVID-19	18 (42.9)	5 (27.8)	8 (44.4)	5 (83.3)	0.058
Signs and symptoms					
Gastrointestinal symptoms	32 (76.2)	14 (77.8)	12 (66.7)	6 (100)	0.260
**Mucocutaneous symptoms**	**36 (85.7)**	**16 (88.9)**	**18 (100)**	**2 (33.3)**	**0.000**
**Cardiovascular symptoms**	**37 (88.1)**	**17 (94.4)**	**17 (94.4)**	**3 (50.0)**	**0.006**
Respiratory symptoms	18 (42.9)	7 (38.9)	9 (50.0)	2 (33.3)	0.716
Neurological symptoms	13 (31.0)	7 (38.9)	4 (22.2)	2 (33.3)	0.571
Renal symptoms	8 (19.0)	4 (22.2)	3 (16.7)	1 (16.7)	0.909
Other symptoms *	3 (7.1)	2 (11.1)	1 (5.6)	0 (0)	0.638
MIS-C phenotype					
**Kawasaki-like disease**	**31 (73.8)**	**15 (83.3)**	**14 (77.8)**	**2 (33.3)**	**0.047**
MIS-C with shock;	5 (11.9)	0 (0)	4 (22.2)	1 (16.7)	0.116
**Undifferentiated MIS-C**	**6 (14.3)**	**3 (16.7)**	**0 (0)**	**3 (50.0)**	**0.007**
Echocardiography and electrocardiography at admission					
Abnormal echocardiogram	30 (71.4)	13 (72.2)	15 (83.3) ^a^	2 (33.3) ^a^	0.064
Abnormal ECG	22 (52.4)	9 (50.0)	10 (55.6)	3 (50.0)	0.943
Laboratory test results at admission (normal range), peak value: median (IQR)					
White blood cell count (4.5–12.0 × 10^9^/L; *n* = 42)	15.4 (10.9–20.1)	16.8 (11.7–25.1)	14.5 (10.9–17.7)	15.1 (8.2–16.8)	0.801
**Absolute lymphocyte count** **(1.5–6.8 × 10^9^/L; *n* = 42)**	**2.2** **(1.2–4.7)**	**3.9** **(2.0–9.2)**	**1.3** **(0.9–3.1)**	**1.3** **(0.8–4.6)**	**0.008**
Absolute neutrophil count (1.5–7.5 × 10^9^/L; *n* = 42)	10.9 (7.7–14.1)	11.1 (8.1–14.1)	11.0 (7.7–15.5)	9.3 (7.0–14.8)	0.641
Platelets (140–450 × 10^9^/L; *n* = 42)	352.5 (137.3–612.8)	442.5 (138.0–657.0)	371.0 (137.3–570.0)	162.5 (100.3–367.0)	0.169
C-reactive protein (<5 mg/L; *n* = 42)	158.6 (100.7–228.5)	148.7 (115.9–230.9)	170.9 (61.6–218.5)	165.5 (87.5–256.4)	0.801
Procalcitonin (<0.05 ng/mL; *n* = 41)	3.4 (1.6–7.4)	3.5 (1.4–7.1)	3.4 ^b^ (1.4–5.1)	12.0 ^b^ (1.6–31.0)	0.983
Interleukin–6 (<5.9 ng/L; *n* = 23)	103.0 (28.6–238.0)	142.0 (34.8–395.8)	46.0 (20.5–190.8)	49.3 (49.3–49.3)	0.142
**Troponin I** **(<16 ng/L; *n* = 40)**	**12.5** **(2.0–70.3)**	**2.5** **(1.0–13.0)**	**50.0** **(6.0–225.5)**	**14.0** **(4.0–439.0)**	**0.036**
B-type natriuretic peptide (<100 ng/L; *n* = 35)	207.3 (71.1–960.2)	113.4 (17.5–794.9)	611.4 ^c^ (88.5–965.5)	141.9 ^c^ (47.1–434.8)	0.276
**Ferritin** **(7–140 μg/L; *n* = 41)**	**375.5** **(252.7–501.7)**	**289.0** **(212.6–386.9)**	**384.6** **(252.4–846.6)**	**481.6** **(361.5–1247.8)**	**0.038**
D-Dimer (45–280 μg/L; *n* = 42)	2102.5 (1283.8–3170.0)	2007.5 (1502.5–2721.3)	2142.5 (907.5–4906.3)	1990.0 (1113.8–7519.5)	0.801
Albumin (38–54 g/L, *n* = 34)	29.64 (25.6–34.3)	28.4 (26.5–35.5)	29.0 (24.4–34.2)	32.7 (29.5–38.4)	0.567
Estimated glomerular filtration rate (*n* = 41)	108.4 (83.5–123.8)	114.4 (95.5–129.6)	110.3 (88.1–121.7)	56.2 (42.9–94.0)	**0.012**
Management					
Intravenous immunoglobulin	39 (92.9)	17 (94.4)	17 (94.4)	5 (83.3)	0.638
Glucocorticoids	34 (81.0)	13 (72.2)	16 (88.9)	5 (83.3)	0.458
Oxygen therapy	11 (26.2)	3 (16.7)	7 (38.9)	1 (16.7)	0.284
Vasoactive drugs	5 (11.9)	0 (0)	4 (22.2)	1 (16.7)	0.116
ICU treatment	28 (66.7)	11 (61.1)	13 (72.2)	4 (66.7)	0.792

* arthralgia, myalgia. ^a^ *p* = 0.038; ^b^ *p* = 0.018; ^c^ *p* = 0.038. Significant differences (*p* < 0.05) among age groups (<5 Years; 5–11 Years; ≥12 Years) are bolded.

**Table 2:** Initial and early follow-up echocardiographic findings of MIS-C patients.

Echocardiographic Findings	Initial Findings, *n* (%)	Early Outcome on Discharge, *n* (%)	*p*-Value
Left ventricular ejection fraction < 55%	11 (26.2)	0 (0)	N/A
Coronary artery dilation	1 (2.4)	0 (0)	N/A
Mitral valve regurgitation	13 (31.0)	4 (9.5)	**0.012**
Tricuspid valve regurgitation	13 (31.0)	3 (7.1)	**0.013**
Pericardial effusion	27 (64.3)	6 (14.3)	**0.000**

*p* values less than 0.05 are bolded; N/A—not applicable.

**Table 3:** Comparison between MIS-C patients admitted or not admitted to the intensive care unit (ICU).

	Non-ICU Patients	ICU Patients	*p*-Value
Counts (%)	14 (33.3)	28 (66.7)	
<5 years	7 (50.0)	11 (39.3)	0.369
5–11 years	5 (35.7)	13 (46.4)	0.373
≥12 years	2 (14.3)	4 (14.3)	0.666
Male sex	10 (71.4)	21 (75.0)	0.541
Comorbidities	3 (21.4)	6 (21.4)	1.000
**A known history of symptomatic COVID-19**	**3 (21.4)**	**15 (53.6)**	**0.047**
Signs and symptoms			
Gastrointestinal symptoms	12 (85.7)	20 (71.4)	0.267
Mucocutaneous symptoms	12 (85.7)	24 (85.7)	1.000
**Cardiovascular symptoms**	**10 (71.4)**	**27 (96.4)**	**0.035**
**Respiratory symptoms**	**3 (21.4)**	**15 (53.6)**	**0.047**
Neurological symptoms	4 (28.6)	9 (32.1)	0.553
Renal symptoms	2 (14.3)	6 (21.4)	0.457
Other symptoms *	2 (14.3)	1 (3.6)	0.254
MIS-C phenotype			
Kawasaki-like disease	11 (78.6)	20 (71.4)	0.459
MIS-C with shock	0 (0)	5 (17.9)	0.116
Undifferentiated MIS-C	3 (21.4)	3 (10.7)	0.311
Echocardiography and electrocardiography at admission			
Abnormal echocardiogram	9 (64.3)	21 (75.0)	0.353
Abnormal ECG	7 (50.0)	15 (53.6)	0.543
Laboratory test results, peak value: median (IQR)			
White blood cell count (n = 42)	15.0 (10.2–18.0)	15.4 (11.4–21.7)	0.545
Absolute lymphocyte count (n = 42)	1.9 (1.3–3.6)	2.2 (1.2–5.5)	0.901
Absolute neutrophil count (n = 42)	10.2 (7.6–13.1)	11.0 (8.8–15.2)	0.289
Platelets (n = 42)	457 (236.8–587.3)	252 (127.5–614.3)	0.712
**C-reactive protein (n = 42)**	**97.0 (28.6–145.1)**	**196.8 (138.2–249.8)**	**0.000**
**Procalcitonin (n = 41)**	**1.5 (0.7–3.1)**	**4.0 (2.6–15.4)**	**0.003**
Interleukin-6 (n = 23)	49.2 (24.6–298.6)	117.5 (28.8–245.8)	0.656
Troponin I (n = 40)	3.0 (1.3–27.0)	13.5 (2.3–79.3)	0.466
**B-type natriuretic peptide (n = 35)**	**82.4 (10.7–418.8)**	**351.9 (95.0–965.4)**	**0.041**
Ferritin (n = 41)	313.5 (202.6–382.3)	390.6 (266.6–635.9)	0.125
D-Dimer (n = 42)	1900 (600.0–2302.5)	2190 (1367.5–5058.8)	0.316
**Albumin (n = 34)**	**38.3 (34.0–40.0)**	**27.6 (24.4–32.0)**	**0.000**
Estimated glomerular filtration rate (n = 41)	110.7 (92.1–126.1)	103.8 (103.8–123.0)	0.118
Management			
**Intravenous immunoglobulin**	**11 (78.6)**	**28 (100)**	**0.032**
Glucocorticoids	11 (78.6)	23 (82.1)	0.543
**Oxygen therapy**	**0 (0)**	**11 (39.3)**	**0.005**
Vasoactive drugs	0 (0)	5 (17.9)	0.116

* arthralgia, myalgia; Significant differences (*p* < 0.05) between Non-ICU and ICU patients are bolded.

## Data Availability

The data set could be available with reasonable request presenting data analysis plan to the corresponding author.
